# Investigation of the Effects of Intra-articular Tranexamic Acid on Intact Cartilage Tissue and Cartilage Formation in Osteochondral Defects of the Rabbit Knee: An Experimental Study

**DOI:** 10.7759/cureus.14873

**Published:** 2021-05-06

**Authors:** Fevzi̇ Bi̇ri̇si̇k, Serkan Bayram, Mehmet Çakmak, Evşen Apaydın, Ali Erşen

**Affiliations:** 1 Orthopaedics and Traumatology, Istanbul Training and Research Hospital, Istanbul, TUR; 2 Orthopaedics and Traumatology, Istanbul University School of Medicine, Istanbul, TUR; 3 Orthopaedics and Traumatology, University College of Medical Sciences, Kirsehir, TUR; 4 Pathology, Istanbul University School of Medicine, Istanbul, TUR

**Keywords:** tranexamic acid, osteochondral defects, intact cartilage, international cartilage repair society

## Abstract

Background

In this study, the effects of tranexamic acid (TXA) and saline on intact cartilage and the recovery of experimental osteochondral lesions following microfracture in a rabbit model were compared.

Methods

Twenty adult rabbits were divided into four groups (1A, 1B, 2A, and 2B) based on with or without TXA use and microfracture. In addition, these groups were categorized into two different subgroups based on the use of TXA in Groups 1 and 2 (Groups A and B). Full-thickness cartilage defects were created on the weight-bearing surface of the medial femoral condyles unilaterally in Group 2 for the effect of TXA or saline on healthy cartilage tissue while a repetitive injection was applied in Group 1 for the effect of TXA or saline on intact cartilage. A single dose of 10 mg/kg TXA was injected into the knee joints of Group A and 10 mg/kg 0.9% saline solution injected in Group B for three consecutive days. All animals were sacrificed for the extraction of the medial condyles for histologic evaluation eight weeks after surgery. The International Cartilage Repair Society (ICRS) II scoring system was used for histologic evaluation.

Results

No complications or adverse effects related to surgery were observed in all rabbits. All ICRS II parameters were similar in the TXA and saline solution groups in the intact cartilage group except for chondrocyte clustering, formation of a tidemark, subchondral bone abnormalities, and mid/deep zone assessment. Moreover, these parameters were higher in the saline solution group in the cartilage group, but no significant difference was observed in the TXA group in the intact cartilage group. All ICRS II parameters were higher in the saline solution group than in the TXA group in the microfracture group, but no significant difference was observed in the TXA group in the microfracture group except for inflammation, which was similar in the TXA and saline solution groups in the microfracture group.

Conclusion

We found that intra-articular TXA administration did not have a negative impact on healthy cartilage tissue and cartilage transformation and proliferation as compared to the saline infusion.

## Introduction

Tranexamic acid (TXA) is an antifibrinolytic agent that prevents plasmin formation, thus inhibiting the breakdown of fibrin clots [1-2]. Previous studies have reported the systemic and topical use of TXA for reducing blood loss and the need for blood transfusions [3-4]. Although TXA is used in major surgeries when higher blood loss is expected, such as hip arthroplasty [5], it may also be expansively used in minimally invasive procedures, such as knee arthroscopy, to improve the surgical approach and functional outcome by reducing postoperative bleeding and swelling [6]. TXA can be administered intravenously, intra-articularly, or orally in the setting of surgery. Although intravenous TXA is widely used, it may also be associated with postopera­tive seizures and increased thromboembolic events [7]. Therefore, intra-articular application is preferred for reducing blood loss and the need for blood transfusions, which has been increasing over the past several years [8].

Although the intra-articular application of TXA is commonly practiced due to its lower rates of systemic effects, the potential damage on articular cartilage following its intra-articular use is yet to be investigated. Tuttle et al. reported that TXA was cytotoxic to bovine and murine cartilage at the dose of 100 mg/mL, but the safety cutoff dose was found to be 25 mg/ml for TXA [9-10]. However, no study has compared the effect of the intra-articular application of TXA on intact cartilage tissue and cartilage formation in osteochondral defects.

In this study, the effects of TXA and saline on intact cartilage and the recovery of experimental osteochondral lesions following microfracture in a rabbit model were compared.

## Materials and methods

Twenty-four male, adult, white New Zealand rabbits (18-24 months old and 3-3.50 kg) were provided by Istanbul University Aziz Sancar Institute of Experimental Medicine Laboratories (Istanbul, Turkey) and were maintained and housed at the same center of Istanbul University in accordance with the regulations set forth by the Office of Protection of Research Subjects. The study was performed under a protocol approved by the Istanbul University Istanbul Faculty of Medicine Animal Research Committee (2017/20385).

Study design and surgical procedure

**Figure 1:**
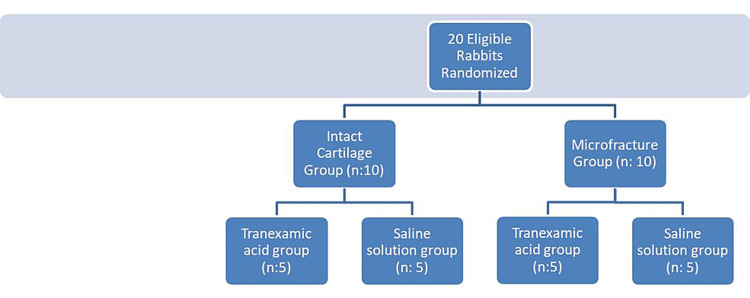
Flow chart of the groups

Twenty adult rabbits were randomly divided into two groups based on whether they were with or without a microfracture (Groups 1 and 2). In addition, these groups were categorized into two different subgroups based on the use of TXA in Groups 1 and 2 (Group 1-A and 1-B; Group 2-A and 2-B) (Figure-1).

**Figure 2:**
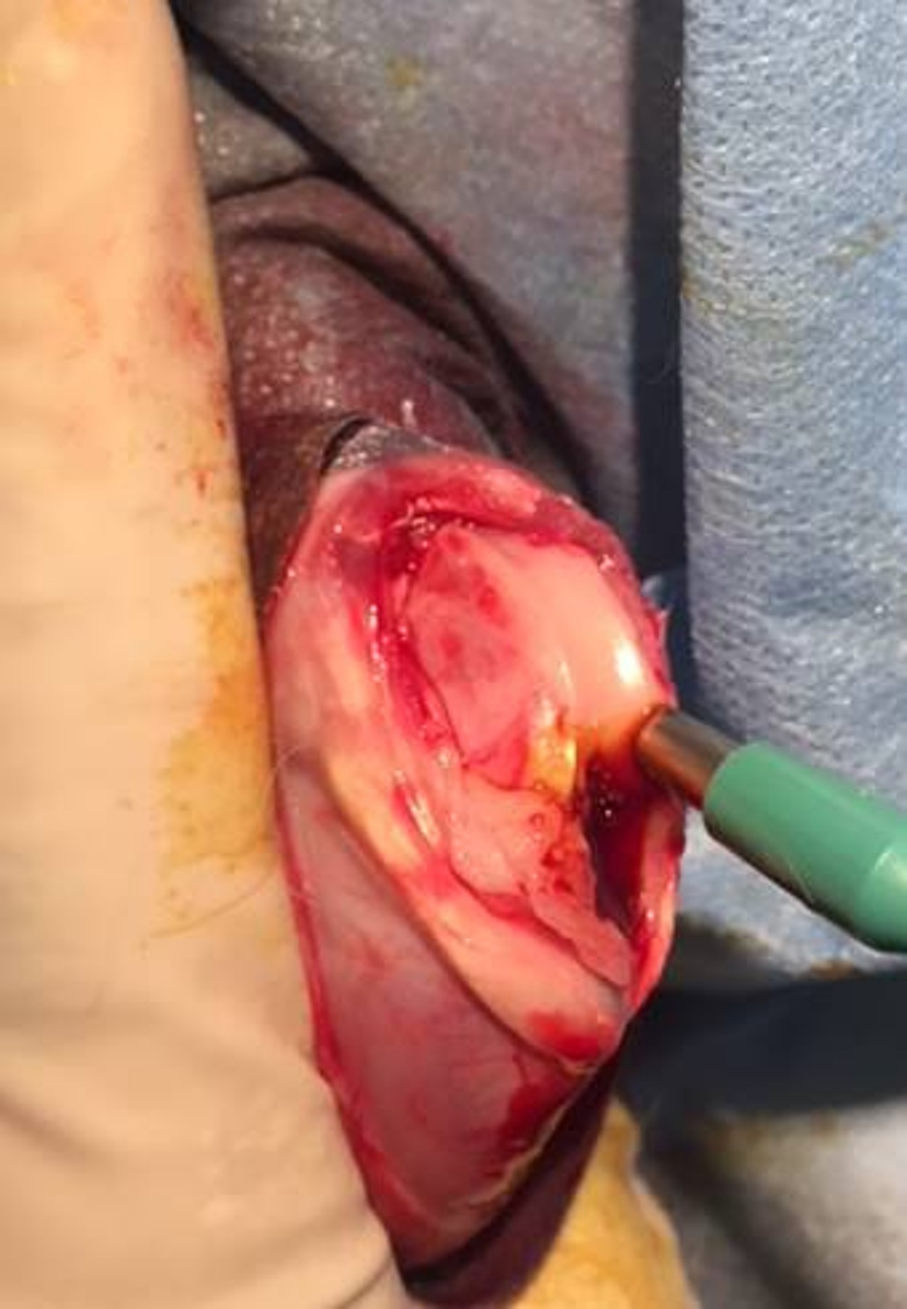
Cylindrical osteochondral defects were created at the weight-bearing surfaces of the medial condyles of the femur, with a size of 4 mm wide and 2 mm deep, by using a dermal biopsy punch

All rabbits were treated unilaterally in Group 2. A single orthopedic surgeon with previous experience in animal studies performed all the procedures. The rabbits were premedicated with buprenorphine 30 minutes before the procedure. The rabbits were anesthetized using 1 mL of xylazine and 1.0-1.2 mL of ketamine intramuscularly. Anesthesia was maintained with 1%-4% isoflurane. An aseptic technique was used for all surgical procedures. Preoperatively, all animals received antibiotic prophylaxis (50 mg/kg cephalosporin sodium) subcutaneously. The right knees of the rabbits were shaved, and the surgical area was cleared of the hair, cleaned with betadine or alcohol, and closed with surgical dressing. A medial parapatellar arthrotomy was performed after the standard anterior midline skin incision, and the patella was everted laterally. Cylindrical osteochondral defects were created at the weight-bearing surfaces of the medial condyles of the femur, with a size of 4 mm wide and 2 mm deep, by using a dermal biopsy punch (Figure 2).

The guidelines of the American Society for Testing and Materials International indicate that the critical size of a chondral defect in a rabbit is a diameter of 3 mm [11]. Watertight closure of the joint capsule was done with 3-0 Vicryl® and skin closure was applied with 3-0 Monocryl®. Following the closure of the joint capsule, a single dose of 10 mg/kg TXA was injected into the knee joints of Group A and 10 mg/kg 0.9% saline solution injected in Group B for three consecutive days. In Group 1, a single dose of 10 mg/kg TXA was intra-articularly injected in Group A and 10 mg/kg 0.9% saline solution in Group B for three consecutive days. All animals received antibiotic prophylaxis (cephalosporin sodium) postoperatively for one day. All rabbits were administered with postoperative non-steroid anti-inflammatory drugs for three to five days as required. The animals were maintained at a temperature of 20°C ± 3°C with a relative humidity of 40%-60% and a photoperiod of 12/12 h (light and dark). All the rabbits were allowed to move freely and housed in a stainless steel cage with a bottom grid. The animals were fed with a pellet diet and had continuous access to tap water.

Furthermore, all animals were sacrificed in accordance with the Institutional Animal Care and Use Committee Protocol with high-dose sodium pentobarbital (>200 mg/kg) for the extraction of the medial condyles for histologic evaluation eight weeks after surgery.

Historical evaluation

A macroscopic examination of the implantation site was performed by observing the appearance of the tissue in situ and photographic documentation. The acquired specimens of the distal femur were embedded in 10% formalin for one week and decalcified in 10% ethylenediaminetetraacetic acid (EDTA)-buffered saline solution for five days. Moreover, the samples were embedded in paraffin blocks and cut into 4 μm sections. For histopathological evaluation, hematoxylin-eosin staining was used for the evaluation of the general morphology and safranin O for the evaluation of proteoglycan content. All sections were blindly scored by a single pathologist according to the International Cartilage Repair Society (ICRS) II scoring system, which had 14 grading criteria [12].

The ICRS II scoring system consists of 14 parameters, including tissue morphology (viewed under polarized light), matrix staining (metachromasia), cell morphology, chondrocyte clustering (four or more grouped cells), surface architecture, basal integration, formation of a tidemark, subchondral bone abnormalities/marrow fibrosis, inflammation, abnormal calcification/ossification, vascularization (within the repaired tissue), surface/superficial assessment, mid/deep zone assessment, and overall assessment. Each ICRS II parameter can range from 0 to 100. A higher score indicates better quality cartilage. Hence, a score of 0 was assigned for properties considered indicative of poor quality cartilage and 100 for good-quality cartilage. All parameters were scored with this method.

Statistical analysis

All statistical analyses were conducted using the Statistical Package for Social Sciences (SPSS®) version 22.0 software (IBM Corp, Armonk, NY). Descriptive statistical methods were used to evaluate study data. The normality of distribution was tested using the Shapiro-Wilk test. Non-normally distributed variables were compared using the Mann-Whitney U test. Moreover, a P-value of <0.05 was regarded as significant.

## Results

No complications or adverse effects related to surgery were observed in all rabbits. The macroscopic evaluation of femoral condyles showed that the defect areas in groups were filled with repair tissue, which is irregular and rough in varying proportions.

**Figure 3:**
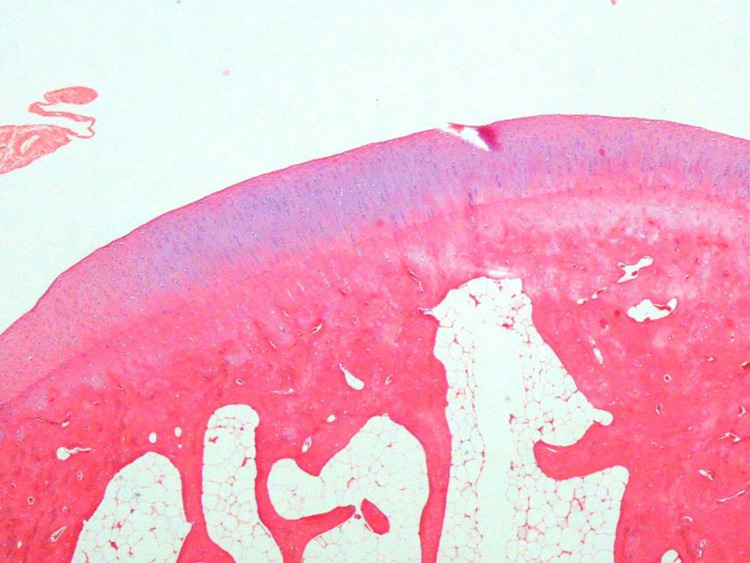
A usual findings were detected in histological examination (Group 1-B) H&E (hematoxylin and eosin) 40x

**Figure 4:**
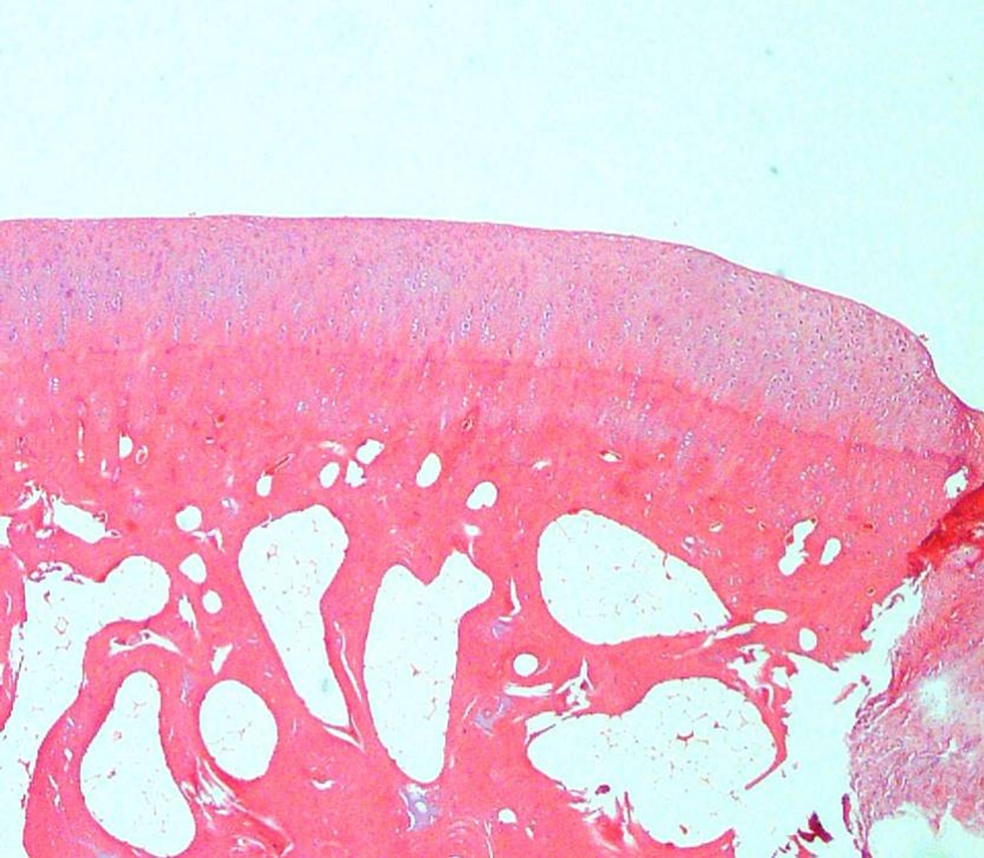
There were no additional noticeable findings related to the defect or drug (Group 1-A) H&E (hematoxylin and eosin) 40x

All ICRS II parameters were similar in the TXA and saline solution groups in the intact cartilage group except for chondrocyte clustering, formation of a tidemark, subchondral bone abnormalities, and mid/deep zone assessment. Moreover, these parameters were higher in the saline solution group in the cartilage group, but no significant difference was observed in the TXA group in the intact cartilage group (Table 1; Figures 3-4).

**Table 1:** Comparison of ICRS II scoring between the intact cartilage groups ICRS: International Cartilage Repair Society

Parameters	Tranexamic Acid Group (Group 1-A)		Saline Solution Group (Group 1-B)		
	Mean ± SD	Min-Max	Mean ± SD	Min-Max	p value
Tissue morphology	100		100		1
Matrix staining	100		100		1
Cell morphology	100		100		1
Chondrocyte clustering	94 ± 8	80-100	96 ±4.8	90-100	0.796
Surface architecture	100		100		1
Basal integration	100		100		1
Formation of a tidemark	92 ± 11.6	70-100	96 ± 8	80-100	0.475
Subchondral bone abnormalities	94 ± 8	80-100	94 ± 4.8	90-100	0.734
Inflammation	100		100		1
Abnormal calcification/ossification	100		100		1
Vascularization	100		100		1
Surface/superficial assessment	100		100		1
Mid/deep zone assessment	94 ± 4.8	90-100	98 ± 4	90-100	0.394
Overall assessment	100		100		1

**Figure 5:**
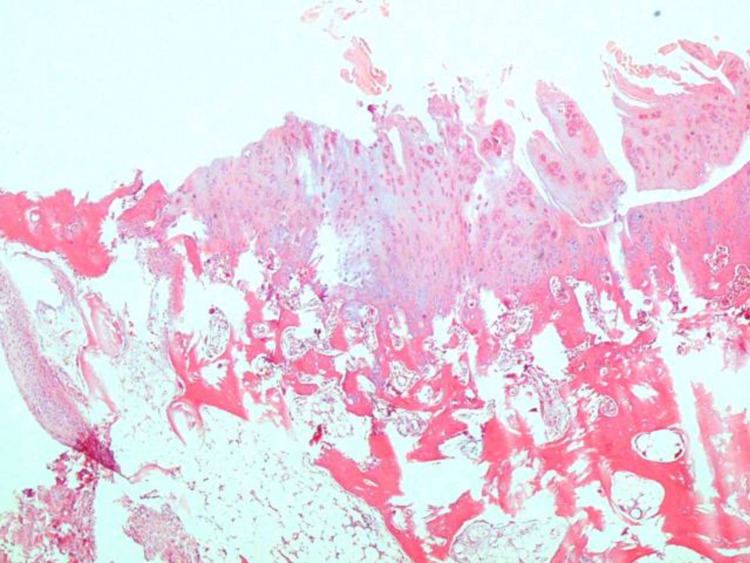
Major irregularity, loosening, and disruptions were seen on the surface architecture. Integration with the subjacent bone was poor. Abnormal ossification and chondrocyte clustering (arrows) were also noted (Group 2-B) H&E (hematoxylin and eosin) 40x, 100x

**Figure 6:**
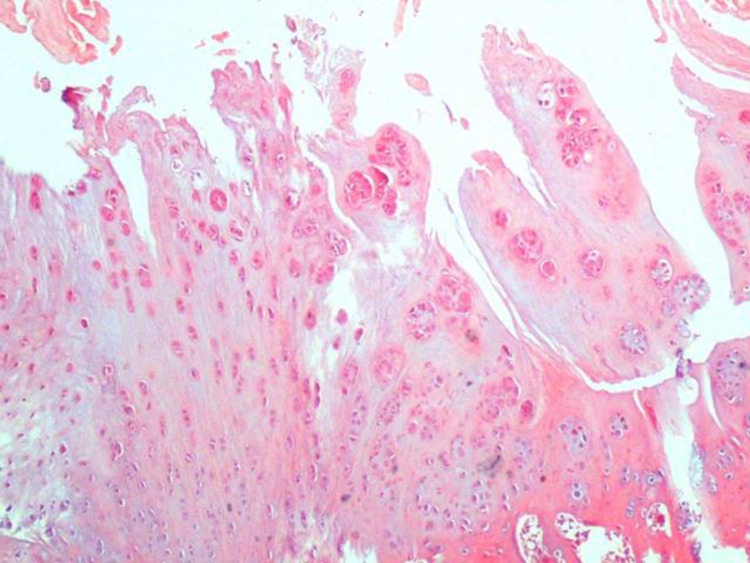
Major irregularity, loosening, and disruptions were seen on the surface architecture. Integration with the subjacent bone was poor. Abnormal ossification and chondrocyte clustering (arrows) were also noted (Group 2-B) H&E (hematoxylin and eosin) 40x, 100x

**Figure 7:**
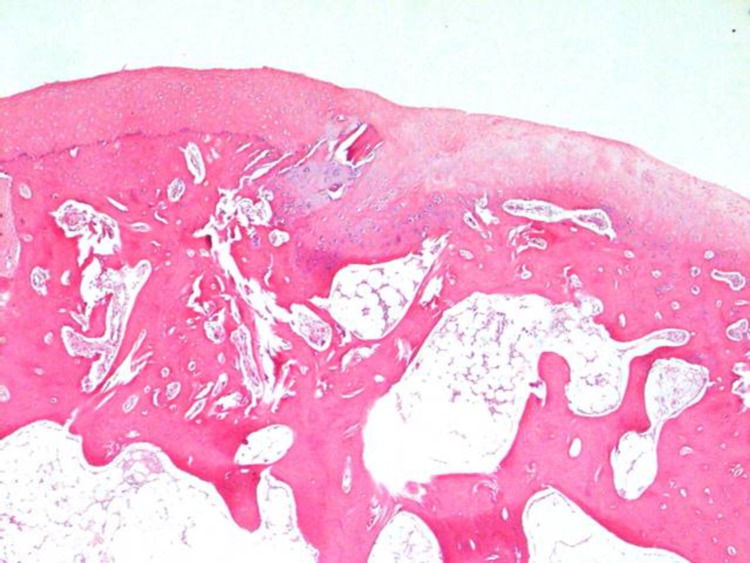
Smooth surface architecture and almost full basal integration along the whole cartilage bone interface were observed. The cells associated with the hyaline cartilage were composed of mostly round/oval cells. The tidemark formation between the radial zone and the calcified cartilage was noted (arrows) (Group 2-A). H&E (hematoxylin and eosin) 40x, 100x

**Figure 8:**
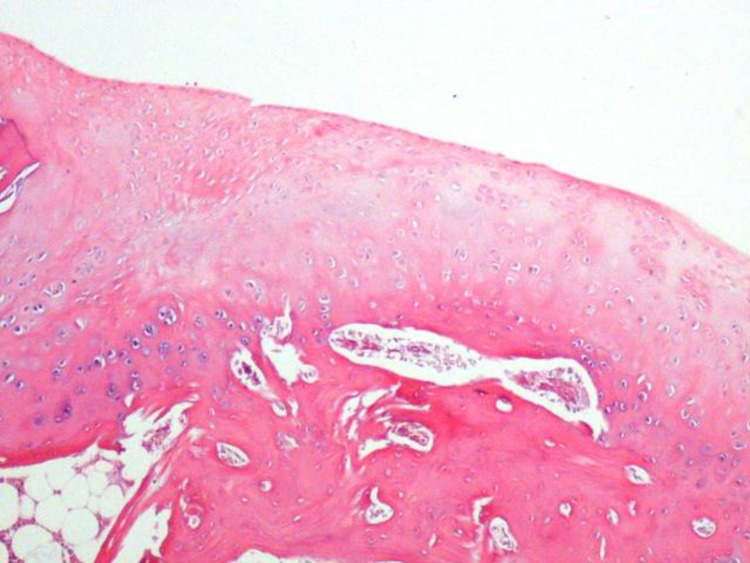
Smooth surface architecture and almost full basal integration along the whole cartilage-bone interface were observed. The cells associated with the hyaline cartilage were composed of mostly round/oval cells. The tidemark formation between the radial zone and the calcified cartilage was noted (arrows) (Group 2-A). H&E (hematoxylin and eosin) 40x, 100x Since the organization and quality of regenerative cartilage tissue formed after a microfracture may differ between groups, the difference in appearance in the cross-section is due to this. Basically, the histopathological formation was similar in the two samples.

All ICRS II parameters were higher in the saline solution group than in the TXA group in the microfracture group, but no significant difference was observed in the TXA group in the microfracture group except for inflammation, which was similar in the TXA and saline solution groups in the microfracture group (Table 2; Figures 5-8.

**Table 2:** Comparison of ICRS II scoring between the microfracture group ICRS: International Cartilage Repair Society

Parameters	Tranexamic Acid Group (Group 2-A)		Saline Solution Group (Group 2-B)		
	Mean ± SD	Min-Max	Mean ± SD	Min-Max	p value
Tissue morphology	42.5 ±15	30-60	46.6 ± 16.3	30-70	0.658
Matrix staining	52.5 ± 20	30-80	56.6 ± 32	20-100	0.915
Cell morphology	76.2 ± 9.4	70-90	86.6 ± 12.1	70-100	0.157
Chondrocyte clustering	60 ± 32	10-100	66.6 ± 17	50-100	0.857
Surface architecture	62.5 ± 18	50-90	71.6 ± 22	30-100	0.513
Basal integration	67.5 ± 12.5	50-80	70 ± 25	40-100	0.745
Formation of a tidemark	41.2 ± 10.3	30-60	46.6 ± 20	20-70	0.745
Subchondral bone abnormalities	61 ± 21	30-80	61.6 ± 20	40-90	0.914
Inflammation	100		100		1
Abnormal calcification/ossification	87.5 ± 25	50-100	91.6 ± 20	50-100	0.759
Vascularization	37.5 ± 17	20-60	40 ± 36	0-100	0.829
Surface/superficial assessment	67.5 ± 15	50-80	70 ± 27	20-100	0.666
Mid/deep zone assessment	60 ± 11	50-70	65 ± 17	40-80	0.511
Overall assessment	60 ± 14	40-70	65 ± 22	30-90	0.591

## Discussion

Although the intra-articular use of TXA is thought to have a less systemic effect, its effect on articular cartilage continues to be investigated. In this study, we investigated the effect of TXA on intact cartilage and the recovery of experimental osteochondral lesions following microfracture in a rabbit model. Based on the results, TXA was observed to have no significant side effects either on intact cartilage tissue and the recovery of experimental osteochondral lesions following microfracture based on the ICRS II scoring system.

One of the primary goals of the previous studies was to investigate the effect of topical TXA on native cartilage. Tuttle et al. investigated the in vitro effects of TXA on bovine cartilage and murine chondrocytes [9]. In that study, they administered TXA in three doses (25, 50, and 100 mg/mL) and found that the 50 mg/mL and 100 mg/mL doses of TXA had a cytotoxic effect on the chondrocytes and damaged the cartilage. However, a 25 mg/mL dose of TXA may be an effective and safe dose for the intra-articular use of TXA in native joints. In another study, Parker et al. investigated the side effects of TXA on chondrocytes and determined a safe dose for its intra-articular use in clinical practice [10]. In that study, they found that no cytotoxic effect was observed when in vitro chondrocytes were exposed to TXA in a concentration that could be expected following intravenous administration, whereas cytotoxicity was observed in vitro when the chondrocytes were exposed to a higher concentration following intra-articular administration. In the present study, we investigated the effect of TXA on either intact cartilage and the recovery of osteochondral defects by comparing the saline solution infusion that was evaluated based on the ICRS II scoring system. Furthermore, it is found that the administration of intra-articular TXA did not have a detrimental effect different from saline infusion on the architecture of articular cartilage.

The other main subject is the effect of intra-articular TXA on the recovery of experimentally induced osteochondral defects. Avki et al. investigated the effects of TXA on the recovery of full-thickness articular cartilage defects, wherein 2 mm diameter osteochondral defects were made in the rabbits and intra-articular TXA was administered intramuscularly every other day for two to four weeks [13]. In that study, they found that TXA treatment had no side effects on the final quality of osteochondral reparative tissue but may shorten the modulation to fibrocartilage tissue. In another study, Degirmenci et al. evaluated the effect of intra-articular TXA application on the recovery of experimentally induced osteochondral defects in rabbits [14]. In that study, the rabbits were divided into two groups: Group 1 (study group) and Group 2 (control group). One ml TXA was injected into the knee joints of the study group, and histological evaluations were performed in all samples based on the Brittberg and O’Driscoll scores. Moreover, they found that the administration of TXA improves the recovery time and tissue stability in osteochondral defects. In the present study, we compared the effects of TXA and saline solution on the recovery of experimentally induced osteochondral defects and found that the saline solution group had higher ICRS II scores but was not significantly different.

One strength of this study is that it is an in vivo study, as most of the previous studies have performed an in vitro cell culture study that evaluated the effects of TXA on cartilage or chondrocytes. Therefore, our study is more useful in clinical practice. Another strength is that we compared the effect of TXA and saline solution on either intact cartilage and the recovery of experimentally induced osteochondral defects. Lastly, this study used the ICRS II scoring system, which was developed for assessing cartilage repair tissue and not used in previous studies on the effect of TXA on cartilage and chondrocytes.

Nonetheless, our study had several limitations. First, the sample size was relatively small. Radiological evaluation was not performed especially in the microfracture group. The use of a 10 mg/kg dose of TXA may be insufficient. In addition, only one scoring system was used for the evaluation of the effect of TXA on cartilage. Last, only one pathologist evaluated the specimens so no interobserver assessment was performed.

## Conclusions

Tranexamic acid widely is used in orthopedic practice, expecting beneficial effects. In our study, we performed an in vivo study evaluating the effect of tranexamic acid on cartilage transformation and proliferation, which was not investigated using the International Cartilage Repair Society II scoring system in the literature. We found that intraarticular tranexamic acid administration in a limited dose did not have a negative impact on healthy cartilage tissue. Finally, we found that intraarticular tranexamic acid administration in a low dose did not have a negative impact on cartilage transformation and proliferation as compared to saline infusion.
